# Median Lithotomy

**Published:** 1878-05

**Authors:** Charles A. Wall


BUFFALO

### Medical and Surgical Journal.

VOL. XVII. MAY, 1878. No. 10.

### Original Communications.

:o:

ART. I.—*Median Lithotomy*, by Charles A. Wall, B. S.

The question as to the best mode in operating for the removal of vesical calculi, has been one agitating the minds of the profession ever since the procedure was rescued from the hands of charlatans, and placed upon the sound basis of a recognized and approved surgical act. Lateral lithotomy has been for centuries, and is today almost exclusively followed, notwithstanding the fact that many other methods have been proposed and strongly advocated by some of the leading writers upon the subject. It is the object of this paper to endeavor to show that too high an estimate has been accorded this operation, and that another has equal, if not superior, claims to our attention. We shall give, in brief, the various operations, mentioning those only which are essentially different, and then the advantages of the median.

The super pubic,* cutting on the gripe and rectal,* have all been assigned to well deserved oblivion and command no notice, being pre-eminently unscientific and without advocates in this enlightened age. They were the children of a time when every one was totally unacquainted with the anatomy of the parts involved, and were, therefore, discarded as more light dawned upon this science;

*These may be employed when very large stones are found, but are not authorized as generally applicable..—Erichsen's Surgery, vol. II, p, 772.

and as the serious nature of any injury that would follow unskillful surgery was revealed, a more exact and correct practice was adopted.

The only operations that remain are those done in the perineum, which naturally divide themselves into two classes,—the lateral, and the median, each having many modifications, conforming in the greater part with its type. Lateral lithotomy is made with section of the prostate, most often to the left of the median line; median lithotomy, on the other hand, is usually made without any such section, the incision only extending to the urethra. We make this the distinguishing characteristic between the two types, and in speaking of them will keep the fact prominently in mind.

The lateral is so well known that it needs no description. A grooved sound, having been introduced through the urethra into the bladder, an incision, beginning near the center of the perineum at an angle of about 30° to the left, is made to it, and the knife is pressed down the groove into the bladder and then carried out in the same plane as the external cut. These few words readily recall all the minutiæ of the operation, and we proceed to mention some of its changes, of which the most important is the bilateral, where two incisions are made in like manner, one to either side. Instead of the last some prefer to nick the neck of the bladder bilaterally with but one external incision; the lateral, however, is not used with dilatation of the urethra.

The median operation, as its name implies, is done in the middle line as follows: A longitudinal cut about an inch and one-half in length is made in the perineum, extending to within half an inch of the rectum and down to a grooved staff in the urethra. A probe is then passed by the side of the staff and the latter withdrawn. The probe serves as a guide to the membranous portion of the urethra, which is now to be slowly and carefully dilated; it can be most safely accomplished by introducing the finger into the bladder, which may be at the same time thoroughly explored, and the size and nature of the stone determined. The finger being withdrawn, the lithotomy forceps are passed in, the stone seized and taken out, gradually enlarging the canal before the calculus, by the gradual enlargement of the forceps as the blades pass over the stone. The varieties of this operation are numerous and differ widely from each other and are in some respects deviations from the prototype. The most important are the median with section of the prostate, medio-lateral, and the medio-bilateral. The incision in the first is carried down and the prostate cut in the same plane as the external wound. Prof. Gross, in his surgery devotes ten lines to median lithotomy, five historical, five descriptive. He says: "It is not difficult to conceive that this operation might answer admirably in cases of small calculus, while it might be very objectionable in large ones, on account of the inadequancy of the wound, which is comparatively diminutive." It is a dangerous procedure from the liability of wounding the rectum and the prostate may be too largely divided. The medio-lateral and medio-bilateral are made, nicking the base of the bladder in a lateral and bilateral direction respectively, the first part following the same line as the median operation. There are many changes in the details of the types, and even in their modifications which are considered by the originators as of prime importance, that we have no space to notice, such as the relative length of the external incision, the exact spot that the cut must begin, the angle of the divergence in the lateral, the size of the division of the prostate, the point where the urethra shall be reached, the form of the staff and shape of the knife used, and the way in which the calculus shall be seized and extracted; all are of minor consequence, and few agree upon any one in every particular. The manner in which the plan is to be carried out, is with the individual surgeon, and is modified as circumstances may present, following it is true one of the authorized operations.

The relative advantages and dangers of either type will determine the advisability of its adoption; and it is only by a careful theoretical study of the parts involved, combined with large clinical researches that the question can finally be settled; theoretically they may be discussed, many facts presenting themselves as undoubtedly true.

The lateral is the quickest to perform, the bladder is most easily reached, and the stone can be seized and removed without difficulty; but it has many dangers, such as the too great section of the prostate, injury to the ejaculatory ducts, and severe if not fatal hæmorrhage from severing large arteries which are so abundantly distributed to the perineum. The last is an objection of such importance that many lithotomists after once encountering it have discarded the operation and selected some other as more desirable. To the median a great number of objections have been urged; abscesses of the neck of the bladder from contusion in withdrawing the forceps, cutting into the rectum, incontinence of urine as a sequela, and lastly, impossibility of the removal of large calculi without rupturing the base of the bladder; the only real advantage allowed it by its opponents being the small wound made in reaching the membranous portion of the urethra; there is, moreover, less danger from arterial lesions. Let us consider these a little more at length. Abscesses are due to violence and force being used, and may be guarded against by allowing the parts time to dilate. Time is of no especial consequence, since the patient is under the influence of an anæsthetic; there is no need to be as expeditious as the older surgeons who prided themselves upon the rapidity with which they could cut for stone; plunging in the knife, introducing the forceps, and extracting the calculus, all in a few seconds of time. That the urethra will expand if time is allowed most will acknowledge, and that the time may be given without prejudice to the patient none can gainsay. In speaking of lithotomy in general Gross says: "Incontinence of urine, consequent upon perineal lithotomy is happily unfrequent." This is as true of median as of lateral; statistics do not carry out the statement that incontinence occurs in more than a small fraction of cases. In support of the last objection, we might bring forward good authority, and it is upon this that most of the oppcsition is laid. If it can not be removed the operation can never hope to gain universal endorsement. Holmes, Ashhurst, Hamilton, Druitt, Bryant and Gross all discourage lithectasy as *contra-indicated* in all cases of large calculi, and as having no counterbalancing merits. If only useful in small calculi then it will never be performed, for a mistake in the diagnosis, as to the size of the stone, would render a change of plan necessary. We think that this vital objection has been successfully overcome by a method that Prof. J. F. Miner has taught and practiced for many years, and which he calls lithotrity through the perineal section. Attention has often been called to this before, and Dr. W. T. Briggs also mentions it in a paper read at Chicago last year, upon 'Medio-Bilateral Lithotomy,'* and although he says that in his opinion no stone should be removed entire through the pelvic outlet, which will require for its extraction a sufficient amount of violence to bruise or lacerate the perineal tissue, and any stone too large for medio-lateral is too large for the lateral or bilateral, still he brings this as *the* objection to the median. In reference to large calculi, he writes : " Other means must be adopted when this complication is met with, and the most rational measure in my opinion is perineal lithotrity." This is as applicable to the median as to the medio-bilateral, and is sufficient answer to the objection long considered as the critical one which if met would bring the operation to the front rank and ensure its adoption. Dr. Miner employs that well known operation called after Allerton, who first brought it prominently before the profession ; and if the calculus is found so large that it can not be removed entire he seizes and crushes it, when the pieces are easily extracted. All débris are washed from the bladder by means of a catheter passed into the urethra. This procedure has been looked upon with fear by surgeons ; why, we can not tell, for to us the danger does not seem greater than in lithotrity per urethra, nor, indeed, so great, since no pieces are left to irritate the surfaces exposed.

The advantages of the median are many. It is the easiest to perform ; all the parts exposed to the knife may be open to the eye of the surgeon, the timid having the opportunity to dissect slowly and carefully until the staff is reached, after which the knife is dispensed with. The parts involved are the least freely supplied with blood vessels ; it seems as though the raphé was intended, by its exemption from important nerves, arteries, or veins, to be the place where lithotomy should be done. Nature laid the affliction, but likewise made this particular line nearly exempt from peril. If care be taken not to go too far forward, thereby wounding the bulb, and the incision restrained one-fourth of an inch before reaching the rectum, no harm can possibly result.

*Transactions of the American Medical Association, vol. 28, p. 529.

No other operation has this advantage, and it is, therefore, the simplest and safest. Practically, little can be said either in favor or against; while over eight thousand cases of lateral lithotomy are recorded and tabulated by Gross, less than five hundred of median can be referred to, in various reports scattered through English and American works. Gross gives a result of 1 in 10.9 for median, lateral being for European operations established as 1 in 8. The statistics carry little weight, because the mortality must depend upon the age, condition and temperament of the patient and the skillfulness of the surgeon. We know of no work equal to Erichsen's surgery for a dispassionate discussion of the relative merits and demerits of the two types, and recommend it as worthy of close study. Although an advocate of the lateral, he gives the median credit for a few advantages and advises its employment in certain classes of cases.

In comparison with lithotrity the median has one advantage; there is but one operation and the bladder is left free from any source of annoyance. The reason that lithotrity is considered so safe may be from the fact that its cases are selected, lithotomy being performed upon the more severe. Every case which is fit for lithotrity would be sure to recover from median lithotomy.

We consider the following as demonstrable facts in reference to median lithotomy:

1. *It is the easiest to an inexperienced surgeon.*
2. *It is the simplest and safest.*
3. *Lithotrity has no advantages over it.*
4. *Applicable to all cases; large calculi must be crushed and removed in pieces.*
5. *The wound is the minimum.*
6. *There is the least loss of blood.*
7. *No danger of urinary infiltration.*
8. *Theoretically it indicates more recoveries; practically statistics are not full enough to prove anything.*

I take great pleasure in reporting the following cases, as illustrative of median lithotomy, as explained above:

Case I.—*Median Lithotomy, Recovery—by Prof. J. F. Miner, M.D.* The patient was a Pole, seven years of age, who had showed all the symptoms of urinary calculus since birth. His parents were poor, ignorant, and had a large family, so that little could be gleaned from them concerning his past history. It was ascertained, that he had been from birth, a cross, fretful, peevish boy, and when he could talk accurately described the symptoms found accompanying stone. Pain was so severe that large quantities of opium were administered with little effect; sudden stoppage of the urine when micturating, and elongation of the prepuce were the most noticeable diagnostic signs. During March, 1877, he came under the treatment of Drs. Dorr and Packwood, who diagnosed vesical calculus and advised an operation. To this the parents consented, and Dr. Miner was asked to operate, which he did April 4, in the presence of a number of physicians and a few medical students. He proceeded about as follows: Dr. Cronyn having brought the patient under the influence of an anæsthetic, a grooved sound was introduced into the urethra as far as possible; but it could not be passed into the bladder, for it came in contact with a hard body beyond which it could not be forced. An incision was now made in the median line until the staff was reached, and a probe having been entered, down the groove and by the side of the stone, into the bladder, Dr. Miner gradually and gently dilated the opening, using the probe as a guide. The sound was withdrawn and the calculus forced into the bladder by the advancing finger. After giving time for the parts to expand, a medium sized pair of lithotomy forceps were entered and the stone seized and extracted. This was done slowly, that the tissues might dilate and not tear. The bladder was again explored and found free. The wound was dressed with warm cloths and the patient put to bed; a small opiate quieted him when restless, but after the first day none was needed. He began to improve immediately, and after two weeks urine passed per urethra; at the end of five weeks he was dismissed from the Hospital, having been well for a number of days. July, 1877, Dr. Dorr informs me that he is better than ever before during his life, and has entire control of his urine. The calculus consisted of two nearly equal parts, joined by an isthmus three-eighths of an inch in diameter and one-eighth of an inch in length. It was two and one-half inches long, and one and one-half inches in diameter. Its weight was three hundred and fifty (350) grains, and it probably consisted of two calculi, which by lying in contact became joined. They were separated in removing, but lay in the forceps in the same relative position as while in the bladder. The calculus being seized in the long diameter was removed as easily as if it had consisted of only one of these parts. The two portions were of nearly equal size, the lower weighing six grains the more; it was also longer and conical in shape, projecting into the urethra, which it completely filled and was the impediment which the sound encountered. The upper part was nearly round, and the axes of the two were inclined at an angle of about one hundred and forty degrees. The calculus was not as large as one previously removed by Dr. Miner from a boy aged 12,* but is of interest from its peculiar shape and its size, the youth of the patient being considered.

Case II.—*Median Lithotomy,—Calculus Crushed,—Recovery—by Prof. J. F. Miner, M. D.* Mr. T——, aged 46, of Bradford, Pa., had suffered for over three years with urinary calculus, and was advised by his physicians to consult Dr. Miner. He was much emaciated; urine constantly dribbled away, and it would often stop suddenly while passing a full stream. For some time he had used large quantities of opium, taking three to five grains at a dose. He showed me a piece of crude opium from which he was accustomed to break off a piece and eat it; the habit, however, was not confirmed. for as soon as the cause was removed he ceased to use it. Nov. 7, 1877, Dr. Miner sounded for stone, but was unable to obtain any confirmatory evidence. On the following day, upon a second examination, a large calculus could be felt without difficulty, and Nov. 10 he operated, in the presence of Drs. McWharf, of Dunkirk, O'Brien, Greene, W. W. Miner and Brush, and twenty medical students. The patient having been anæsthetized, the bladder was reached and the forceps introduced (as in Case I), but instead of removing the calculus entire, it was crushed and the pieces extracted separately, which was easily done, since it was soft although large. After the larger pieces had been taken out,

---

*Buffalo Medical and Surgical Journal. Vol. XIV., No. 9, p. 325.

a catheter was passed into the bladder, per urethra, which was thoroughly washed; all particles remaining being driven out with facility through the perineal incision. The wound was dressed with warm water dressings and the patient placed in bed. He took very little anodyne, and that for a few days only, when he dropped opium entirely and went on to a good recovery. Nov. 19 he returned to his home; he could then control his urine for about two hours, and the wound was nearly healed. He writes lately that he is entirely well and has been attending to business since he went home more attentively than he had been able to do for a year previously. The calculus was soft and light, but of considerable size; it must have been two inches in its smallest diameter; the larger fragments weighed one and one-half ounces, and care was not taken to save any of the pieces which were washed out, some of them being larger than beans. The objection to the median operation that incontinence of urine is apt to follow has been referred to before, and does not seem to be practical but rather theoretical, and more imaginative than real. In this case the result was truly gratifying ; in less than two weeks after the operation the patient was sent home with control of urine for a reasonable length of time. This case is illustrative of those in which the calculus is found too large for removal entire. It is far easier to crush the stone through the incision than through the urethra, and every particle can be washed out. The dangers of lithotomy are not from abscess, incontinence or urinary infiltration, but from previous cystitis, organic kidney disease and general debility, leaving the patient too weak to rally from the prostration already established.

As the Median seems to present most to recommend it, so do we look to see it adopted to the gradual exclusion of all others. The time will be long on its way, but, perhaps it may come, when the median shall hold the enviable position now occupied by the lateral as first choice among operations for stone.

